# The Use of Essential Oil and Hydrosol Extracted from *Cuminum cyminum* Seeds for the Control of *Meloidogyne incognita* and *Meloidogyne javanica*

**DOI:** 10.3390/plants10010046

**Published:** 2020-12-28

**Authors:** Iro Pardavella, Demetra Daferera, Theodoros Tselios, Panagiota Skiada, Ioannis Giannakou

**Affiliations:** 1 Laboratory of Agricultural Zoology and Entomology, Department of Science of Crop Production, Agricultural University of Athens, Iera Odos 75, 11855 Athens, Greece; iro.pardavella@gmail.com (I.P.); stud115071@aua.gr (T.T.); giotask69@gmail.com (P.S.); 2 Laboratory of Chemistry, Department of Food Science and Human Nutrition, Agricultural University of Athens, Iera Odos 75, 11855 Athens, Greece; daferera@aua.gr

**Keywords:** bio-pesticides, nematicidal activity, plant parasitic nematodes control, root-knot nematode management

## Abstract

The essential oil (EO) and hydrosol (HL) isolated from *Cuminum cyminum* (cumin) seeds were evaluated against the root-knot nematodes *Meloidogyne incognita* and *M. javanica*. The efficacy of extracts on the motility, hatching, and survival in soil of second-stage juveniles (J2s), and the activity on egg differentiation were tested. All J2s were paralyzed after immersion in the EO at 62.5 μL/L concentration for 96 h. Encouraging results were recorded using HL equal to or higher than 10% concentration for both *Meloidogyne* species tested. More than 70% paralyzed J2s were recorded after immersion for 48 h, while the percentage was increased to higher than 90% after 96 h of immersion. A clear effect on egg differentiation was observed after immersion in EO or HL. A significant decrease in egg differentiation was revealed at even low concentrations of EO while an evident decrease in egg differentiation was recorded after immersion of eggs in 50% HL dilution. Decreased hatching of *M. incognita* and *M. javanica* J2s was observed with the increase in concentration. The lowest numbers of hatched J2s were recorded when EO was used at 1000 and 2000 μL/L concentrations. A constant reduction in root-knot nematode J2 hatching was observed upon increasing the concentration of HL from 5% to 50%. The EO of *C. cyminum* is characterized by the presence of γ-terpinene-7-al (34.95%), cumin aldehydes (26.48), and α-terpinene-7-al (12.77%). The above constituents were observed in HL following the same order as that observed in EO. The components γ-terpinene (11.09%) and *ο*-cymene (6.56%) were also recorded in EO while they were absent in HL.

## 1. Introduction

Plant parasitic nematodes belonging to the genus *Meloidogyne* are one of the most widespread and destructive groups of plant parasites, and their control is extremely challenging [1]. A large variety of crops worldwide can be infested, and the impact on yield losses has been estimated as a billion euros annually [2]. In recent years, the management of root-knot nematode (RKN) populations in vegetable production was mainly based on the use of chemical nematicides. However, many of the most effective and well-known nematicides, such as methyl bromide and 1,3 dichloropropene, have been phased out due to their adverse effects on the environment. Farmers are obliged or enforced to use other means of management such as cultural practices, crop rotation, resistant cultivars, or rootstocks, while the use of synthetic chemical nematicides is declining. However, the persistence and efficacy of the nematicides still in use are reduced due to enhanced degradation [3,4]. Researchers keep trying to find new molecules effective against plant parasitic nematodes [5,6,7,8,9]. Isolated phytochemicals from different plant species have been used as pesticides themselves or have served as model compounds for synthetic agrochemicals by the industry [10]. Plant-origin essential oils (EOs) and their components have been tested against plant parasitic nematodes [10,11,12]. Volatile organic compounds produced by plants belonging to different botanical families are useful as defense substances against pests and pathogens. They are a complex mixture of mainly terpenoids and a variety of aromatic phenols, oxides, ethers, alcohols, esters, aldehydes, and ketones [13]. Their chemical composition is not consistent among plant species and varieties, or different ratios of substances can be found due to the phenological stages of the plant. There are reports of EOs showing antimicrobial, antifungal [14], antioxidant [15], and insecticidal activities [16,17], antigermination activity in weed species [18], and nematicidal properties [19,20]. Specifically, many EOs and their components have been reported to have efficacy against pinewood (*Bursaphelenchus xylophilus*) and root-knot nematodes (*Meloidogyne* spp.) [10,21,22]. Iacobellis et al. [23] demonstrated the antibacterial activity of *Cuminum cyminum* L. oil against genera responsible for plant or cultivated mushroom diseases. Although there are many studies using EOs, less attention has been paid to the use of hydrosols. Petrakis et al. [24], working with hydrosols (HLs) isolated from three Lamiaceae plants, showed a significant change in *Myzus persicae* behavior. Traka et al. [25] reported that hydrosols derived from *Ocinum basilicum* and *Ruta chalepensis* were effective against *Aphis gossypii* and *Tetranychus urticae*. 

The present study was designed to evaluate the possible nematicidal activity of the EO and HL obtained from *C. cyminum* seeds, as well as to monitor the chemical composition of both extracts.

## 2. Results

### 2.1. Chemical Composition of EO and HL 

All compounds of the EO and HL, identified according to their elution order from the TR-5MS capillary column, the relative retention indices (RRI), and the compounds’ relative percentages in the total composition, are shown in Table 1. Twelve constituents were recorded in the EO, while nine constituents were identified from the HL. *γ*-Terpinen-7-al (34.9%) and cumin aldehyde (26.5%) dominated in the EO, while α-terpinen-7-al (12.8%) and *γ*-terpinene (11.1%) were present to a lesser extent. The HL was characterized by the presence of the same constituents in the same order, i.e., *γ*-terpinen-7-al (42.0%), cumin aldehyde (31.5%) and α-terpinen-7-al (20.9%).

### 2.2. Effect of EO and HL on Second-Stage Juvenile (J2) Motility 

The paralysis caused to *M. incognita* and *M. javanica* J2s by the different concentrations of the EO is presented in Table 2 and Table 3, respectively. The essential oil paralyzed 100% of the J2s of both species after 96 h of immersion at the concentration of 62.5 μL/L. The same level of J2 paralysis remained as the concentration was increased to 500 μL/L. It is noticeable that low J2 paralysis was observed up to the concentration of 31.2 for both species after immersion times of 24 and 48 h, while the recorded paralysis was drastically increased as the concentration was increased to 62.5 μL/L (Table 2 and Table 3). However high numbers of paralyzed *M. incognita* J2s were recorded at the dose of 31.2 μL/L for both experiments after 96 h of incubation (Table 2). 

The paralysis caused to *M. incognita* and *M. javanica* J2s by the different dilutions of the HL is presented in Table 4 and Table 5, respectively. For both nematode species, an increase in the paralyzed J2s was observed with the increase in concentration or exposure time. However, there was variability in paralysis levels between the two experiments, mainly in the lower dilutions for both nematode species. 

### 2.3. Nematostatic Effect of EO on J2s

**Figure 1:**
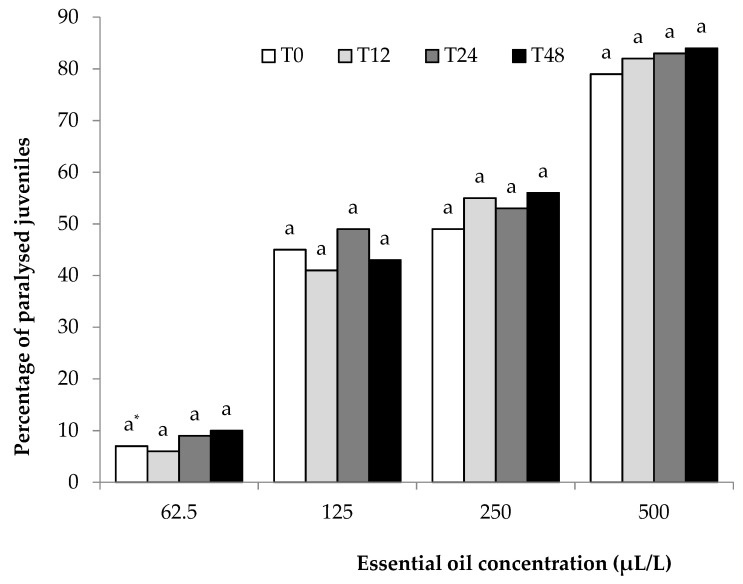
Effect of *Cuminum cyminum* seed essential oil on the motility of *M. incognita* J2s after immersion in test solutions at concentrations of 62.5, 125, 250, and 500 μL/L for 12 h (T0), transferred in water, and counted after 12 (T12), 24 (T24), and 48 (T48) h. * Values for each concentration followed by the same letter are not significantly different according to Tukey’s HSD test at *p* < 0.05.

**Figure 2:**
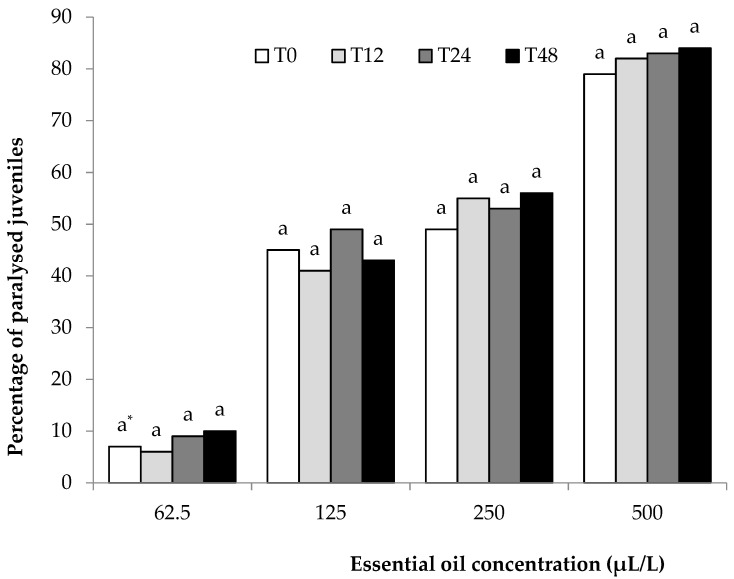
Effect of *Cuminum cyminum* seed essential oil on the motility of *M. javanica* J2s after immersion in test solutions at concentrations of 62.5, 125, 250, and 500 μL/L for 12 h (T0), transferred in water, and counted after 12 (T12), 24 (T24) and 48 (T48) h. * Values for each concentration followed by the same letter are not significantly different according to Tukey’s HSD test at *p* < 0.05.

A nematostatic effect was not observed in the paralyzed J2s. All paralyzed J2s could be considered dead since no J2s regained motility. Furthermore, permanent paralysis (death) was confirmed for *M. incognita* (Figure 1) or *M. javanica* (Figure 2) by maintaining J2s in wells containing water for 12, 24, and 48 h. 

### 2.4. Effect of EO and HL on Egg Differentiation 

**Figure 3:**
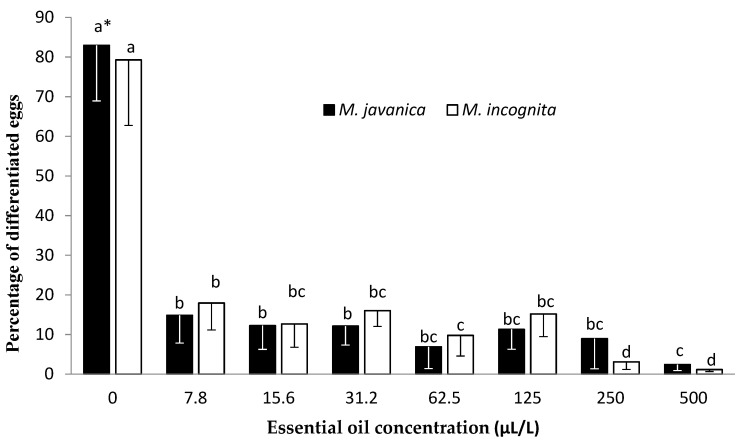
Effect of *Cuminum cyminum* seed essential oil on the differentiation of *M. incognita* and *M. javanica* eggs after immersion in test solutions at concentrations of 0, 7.8, 15.6, 31.2, 62.5, 125, 250, and 500 μL/L for 28 days. * Bars with the same color followed by the same letter are not significantly different according to Tukey’s HSD test at *p* < 0.05.

**Figure 4:**
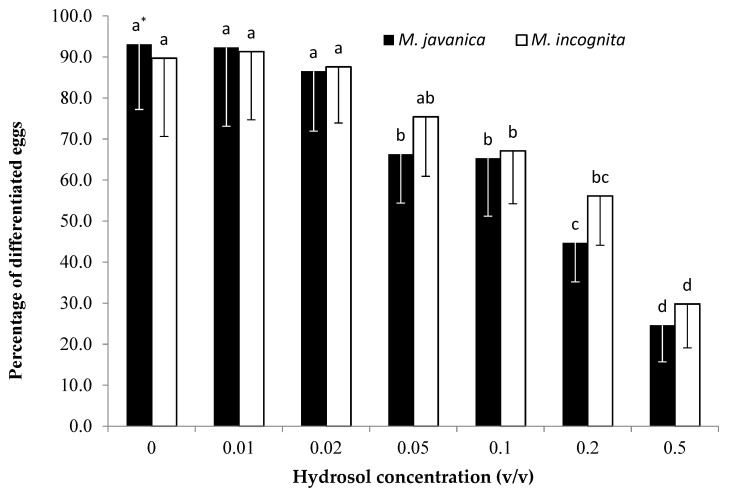
Effect of *Cuminum cyminum* seed hydrosol on the differentiation of *M. incognita* and *M. javanica* eggs after immersion in test solutions at concentrations of 0, 0.01, 0.02, 0.05, 0.1, 0.2, and 0.5 *v/v* for 28 days. Bars with the same color followed by the same letter are not significantly different. * Bars with the same color followed by the same letter are not significantly different according to Tukey’s HSD test at *p* < 0.05.

There was a significant decrease (Figure 3) in the percentage of differentiated eggs (fully developed juvenile) between the control and all concentrations tested. Statistical analysis revealed no significant differences among the different concentrations of the EO for *M. javanica*, apart from the concentration of 500 μL/L, which was significantly lower than the concentrations of 7.8, 15.6, and 31.2 μL/L. The lowest percentages of differentiated eggs were recorded at the concentrations of 250 and 500 μL/L. A significant reduction in egg differentiation was observed when undifferentiated eggs were treated with the lowest EO concentration (7.8 μL/L). When eggs were immersed in HL, a significant decrease in differentiated *M. javanica* eggs was recorded upon increasing the HL dilution to 0.05 *v/v* (Figure 4). A further reduction in egg differentiation was observed upon increasing the HL dilution to 0.2 and 0.5 *v/v*. The lowest percentage differentiation was observed in eggs of both species submerged in a *C. cyminum* dilution of 0.5 *v*/*v* for 28 days. 

### 2.5. Hatching Inhibition as Affected by the Presence of EO and HL 

**Figure 5:**
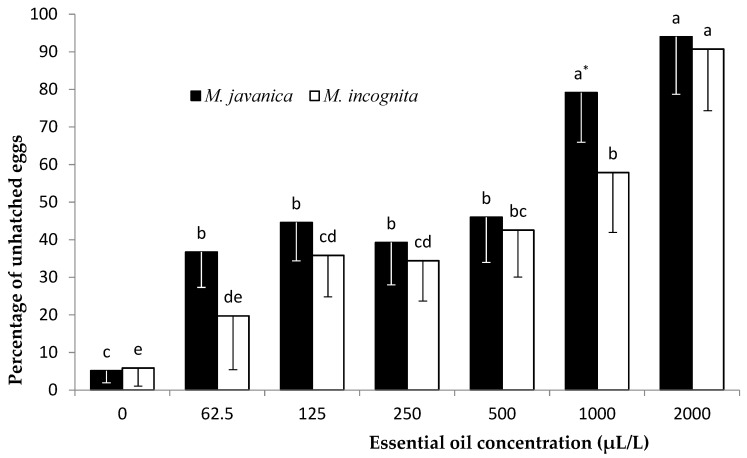
Effect of *Cuminum cyminum* seed essential oil on hatching of *M. incognita* and *M. javanica* J2s after immersion in test solutions at concentrations of 0, 7.8, 15.6, 31.2, 62.5, 125, 250, and 500 μL/L for 35 days. * Bars with the same color followed by the same letter are not significantly different according to Tukey’s HSD test at *p* < 0.05.

**Figure 6:**
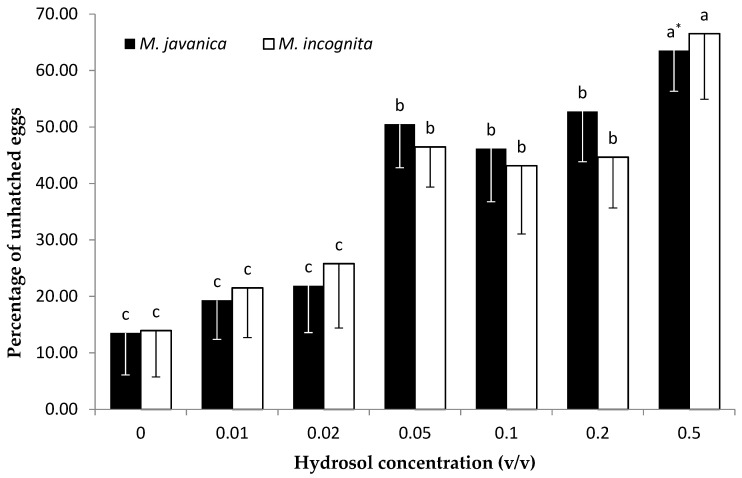
Effect of *Cuminum cyminum* seeds hydrosol on hatching of *M. javanica* and *M. javanica* J2s after immersion in test solutions at concentrations of 0, 0.01, 0.02, 0.05, 0.1, 0.2, and 0.5 *v/v* for 28 days. * Bars with the same color followed by the same letter are not significantly different according to Tukey’s HSD test at *p* < 0.05.

The results of the effect of the EO on hatching inhibition, as means of the two experiments, are presented in Figure 5. The effect of the EO on hatching of J2s was related to different concentrations after 35 days of exposure for both species. A clear reduction in *M. javanica* juvenile hatching was observed as the concentration was increased to 1000 μL/L and 2000 μL/L. No significant differences were recorded among the concentrations in the range from 62.5 to 500 μL/L. Hatching of *M. incognita* J2s decreased with the increase in concentration. The highest number of unhatched eggs was recorded at the dose of 2000 μL/L. For both *Meloidogyne* species, a significant reduction in J2 hatching was observed when the concentration of HL was increased to 0.05 *v/v*. No significant difference was observed for the two lowest HL concentrations (0.01 and 0.02 *v/v*) in comparison with the untreated control (Figure 6). 

### 2.6. Effect of EO and HL on Juveniles in Soil 

**Figure 7:**
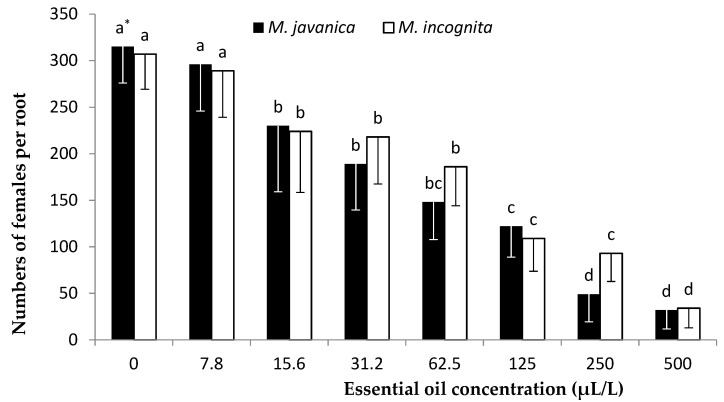
Number of females of *Meloidogyne javanica* and *M. incognita* per tomato root system in soil treated with *Cuminum cyminum* seed essential oil (EO) at concentrations of 0, 7.8, 15.6, 31.2, 62.5, 125, 250, and 500 μL/L and inoculating with 600 J2s. * Bars with the same color followed by the same letter are not significantly different according to Tukey’s HSD test at *p* < 0.05.

**Figure 8:**
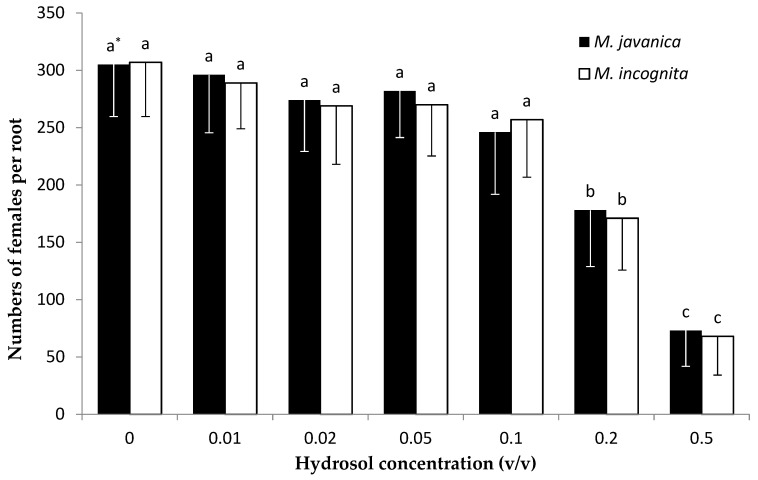
Number of females of *Meloidogyne javanica* and *M. incognita* per tomato root system in soil treated with Cuminum cyminum seed hydrosol at concentrations of 0, 0.01, 0.02, 0.05, 0.1, 0.2, and 0.5 *v/v* and inoculating with 600 J2s. * Bars with the same color followed by the same letter are not significantly different according to Tukey’s HSD test at *p* < 0.05.

A gradual decrease in the number of nematodes per root system was recorded as the concentration of EO was increased from 7.8 to 500 μL/L for both *Meloidogyne* species (Figure 7). The lowest numbers of nematode females were observed at the concentration of 500 μL/L for *M. incognita* and 250 and 500 μL/L for *M. javanica.* No significant differences to the untreated control were recorded for the number of females per root system, whether for *M. incognita* or *M. javanica* at the lowest EO concentration of 7.8 μL/L. Hydrosol dilutions at concentrations up to 0.1 *v/v* showed no significant differences compared to the untreated control for either *M. javanica* or *M. incognita* (Figure 8). A significant decrease was observed after increasing the concentration to 0.2 *v/v* for both *Meloidogyne* species tested, and a further decrease in female numbers per root system was observed after increasing the dilution to 0.5 *v/v*. 

## 3. Discussion

This work is the first study to report the toxic effect of *C. cyminum* extracts (essential oil and hydrosol) against *Meloidogyne incognita* and *M. javanica*. The EO caused a strong nematicidal effect against different biological stages of either *M. incognita* or *M. javanica*. 

Different amounts of terpenes were present in the EO and HL. Monoterpenes occupied almost the whole composition of the essential oil of *C. cyminum.* Hydrocarbon and oxygenated monoterpenes (HMs, OMs) constituted 23.6% and 75.6% of the oil, respectively. The components detected as principles were *γ*-terpinen-7-al, cumin aldehyde, *α*-terpinen-7-al, and *γ*-terpinene, followed by *p*-cymene and *β*-pinene. The above compounds also prevailed (almost 93%) in the essential oil composition of *C. cyminum* from Iran [26]. Quite different was the GC–MS profile obtained from the oil distillated from the dry seeds of *C. cyminum* growing wild in Iran, which exhibited α-pinene, limonene, 1,8-cineole, and linalool (sum 79.5%) as its main compounds [27]. Thymol (40.1%) was abundant in the oil from *C. cyminum* plants. However, it is well known that there are differences in the chemical compositions of the EO obtained from plants grown in different locations [28]. The compounds determined to the composition of *C. cyminum* hydrosol belonged to the group of oxygenated monoterpenes (OMs), and they constituted 99.3% of the oil remaining in water phase. HMs are characterized as nonpolar compounds; thus, their solubility in water is limited, and this can explain why they were not detected in the hydrosol. Terpinen-7-al, cumin aldehyde, and *α*-terpinen-7-al were the dominant compounds of the hydrosol. These compounds were higher than those detected in the EO composition. 

A high percentage of paralyzed J2s was recorded after incubating juveniles for 24, 48, and 96 h. The EO and HL of *C. cyminum* seem to have remarkable nematicidal activity against *M. incognita* and *M. javanica*. There was a drastic increase in J2 paralysis when the concentration was increased from 31.2 to 62.5 μL/L. The juveniles’ paralysis remained constantly high upon a further increase in the concentration to 500 μL/L. Other researchers, working with plant extracts, also reported an effective nematicidal action in J2s. Laquale et al. [29] reported a strong action of EOs isolated from two Italian ecotypes of *Monarda* species. Those essential oils had as constituents, among others, γ-terpinene and *o*-cymene, which are also present in *C. cyminum*. Faria et al. [30], working with isolated EOs from 56 plant samples, found that some of the most successful EOs were those containing 2-undecanone, ascaridol, carvacrol, methyl salicylate, *p*-cymene, and γ-terpinene. Although a straight comparison cannot be made, since essential oils always have different constituents in a different ratio, it seems that *C. cyminum* EO contains some of the most well-known nematicidal compounds, which were responsible for the high efficacy of the tested extract against *M. incognita* and *M. javanica* J2s. An increased number of paralyzed J2s were recorded upon increasing the exposure time (from 24 to 96 h) or the concentration of EO. The hydrosol of *C. cyminum* showed lower nematicidal activity, and J2 paralysis was always less than that observed using EO. 

*C. cyminum* essential oil significantly inhibited egg differentiation. This was noticeable even at the 7.8 μL/L concentration for both nematode species tested. Egg differentiation remained constantly low as the concentration was increased to 250 (*M. javanica*) and 125 μL/L (*M. incognita*). A further concentration increase resulted in an even lower egg differentiation rate. The use of *C. cyminum* HL was also effective in decreasing egg differentiation for both species. When HL was tested on the differentiation of *M. javanica* eggs, a significant decrease was observed upon increasing the concentration to 0.05 *v/v*, while a constant decrease was observed upon increasing the concentration to 0.5 *v/v*. This decrease in egg differentiation correlated with an increase in concentration when *M. incognita* eggs were used, although this was not as obvious as observed in *M. javanica*. 

The egg stage is the most resistant stage in the nematode’s life cycle, due to its three-layer shell consisting of an outer vitelline, a middle chitinous, and an inner lipid layer, providing a remarkable shield of protection. This layer shell is the most important barrier when it comes to breaching and enabling nematicidal activity. Preliminary trials showed that low concentrations (7.8, 15.6, and 31.2 μL/L) did not reveal any effect on hatching and they were not used for this reason, while two higher concentrations (1000 and 2000 μL/L) were included in the trials (Figure 5). EO inhibited *M. javanica* from hatching at concentrations of 62.5 to 500 μL/L, while a further hatching decrease was observed upon increasing the EO concentration to 1000 and 2000 μL/L. Immersion of *M. incognita* eggs in *C. cyminum* EO resulted in a decrease in hatching as the concentration was increased. The lowest concentration tested (62.5 μL/L) did not show any statistical difference compared to the control, while any further increase resulted in a substantial decrease in egg hatching. When HL was used, there were two levels of hatching decrease for both *Meloidogyne* species tested. There was a clear effect on hatching as the concentration was increased to 0.02 *v/v* and a further significant decrease in egg hatching was observed upon increasing the concentration to 0.5 *v/v*. The egg hatching inhibition activity tested on egg masses is an indication of either the EO’s or HL’s ability to penetrate the gelatinous matrix and act on nematode eggs [31]. 

Our results show that significantly low numbers of J2s survived in the soil after the application of *C. cyminum* EO at 250 and 500 μL/L concentrations. The lowest level of nematode infection on tomato roots was observed in the soil treated with EO at 500 μL/L. The use of HL at the concentration of 0.2 *v/v* was effective in reducing the numbers of J2s in soil, and a further significant J2 reduction was observed upon increasing the concentration to 0.5 *v/v*. 

## 4. Materials and Methods

### 4.1. Nematode Populations

The populations of *Meloidogyne incognita* and *M. javanica* were collected from infested tomato greenhouses in Heraklion, Crete and subsequently reared on tomato seedlings (*Solanum lycopersicum* L.) cv. Belladona in a glasshouse at the Agricultural University of Athens, Greece. All the seedlings were maintained in plastic pots in controlled conditions (25 ± 2 °C, 16 h light and 8 h dark). The infestation of seedlings took place when the plants were 4 weeks old, at the four-leaf stage. After 40 days, the infested plants were uprooted, and the roots washed free of soil. Eggs of *Meloidogyne* populations were extracted using 1% sodium hypochlorite solution (Hussey and Barker 1973). Second-stage juveniles (J2s) were hatched in a modified Baermann funnel, where the eggs were placed. The J2s used in all tests were less than 2 days old. 

### 4.2. Isolation of Essential Oil and Hydrosol and Determination of Their Chemical Composition 

Seeds were purchased from a commercial supplier in Athens, Greece. The essential oil was isolated from seeds of *C. cyminum* via hydrodistillation (HD) for 3 h in a 5 L Clevenger-type apparatus. The essential oil was dried with anhydrous magnesium sulfate (MgSO_4_). The hydrosol was collected in a separate glass bottle. A part of it was extracted using diethyl ether in order to isolate the volatile compounds. The organic phase was dried over anhydrous magnesium sulfate, filtered, and reduced to a final volume. Both the essential oil and the organic phase were stored at 18 °C until their analysis. The determination of the chemical composition of the plant isolates was achieved using the gas chromatography–mass spectrometry (GC–MS) technique with a Trace Ultra Gas Chromatographer coupled to a DSQ II Mass Spectrometer (Thermo Scientific), fitted with a TR-5MS (30 m × 0.25 mm × 0.25 μm) capillary column (Thermo Scientific). The injector and MS transfer line temperatures were set at 220 and 250 °C, respectively. Experimental conditions included an oven GC temperature programmed from 60 to 250 °C at a rate of 3 °C/min. Helium was used as the carrier gas at 1 mL/min flow rate. A quantity of 1.0 μL of the diluted samples in the case of EO (1/1000 in acetone, *v*/*v*) or of the organic phase were injected manually in splitless mode. The MS was operated in electrospray ionization (EI) mode at 70 eV, with an ion source temperature of 240 °C, whereas mass spectra were acquired in scan mode for a mass range of 35–400. Tentative identification of the compounds was achieved on the basis of a comparison of their relative retention indices and mass spectra with corresponding data reported in the literature and the instrument’s databases [Adams Book 07, Nist 98, Xcalibur]. The relative retention indices (RRIs) of compounds were determined with reference to the retention times of C_8_–C_24_ *n*-alkanes. Relative percentages of the compounds were obtained electronically from area percentage data.

### 4.3. Effect of EO and Hydrosol on J2 Motility

All in vitro tests were performed in Cellstar^®^ flat-bottom 24-well plates. Solutions of *C. cyminum* essential oil were tested for J2 motility at the doses of 0, 7.8, 15.6, 31.2, 62.5, 125, 250, and 500 μL/L. The essential oil was dissolved in ethanol (Sigma-Aldrich; Italy) and serially diluted in distilled water containing Tween-20 to produce test solutions of the above concentrations. The ethanol and Tween-20 concentrations (1% and 0.2%, respectively) were tested in preliminary tests which showed no effect on nematodes. The hydrosol dilutions (in distilled water) tested for J2 motility were 1% (0.01), 2% (0.02), 5% (0.05), 10% (0.1) 20% (0.2), and 50% (0.5) (*v*/*v*). Distilled water was used as the control. Approximately 50 J2s were used per treatment well in the plates. All plates were covered with aluminum foil and incubated at 26 ± 1 °C. An inverted microscope (Zeiss, Oberkochen, Germany) at 100× magnification was used for the J2 observation after 24, 48, and 96 h of incubation time. The juveniles were scored as motile or paralyzed after 10 s of observation. If there was lack of movement, the J2s were considered paralyzed. Each experiment was conducted twice, and every treatment was replicated five times per trial.

### 4.4. Nematostatic Effect of EO on J2s

*C. cyminum* EO was dissolved in ethanol, and solutions at 0, 62.5, 150, and 500 μL/L concentrations were produced after serially diluting in distilled water containing Tween-20. One hundred milliliters of each solution containing approximately 5000 J2s was transferred to a 250 mL Erlemeyer flask. Oxygen was supplied throughout the experiment using plastic tubes connected to an air pump. Distilled water and water with ethanol and Tween-20 were used as controls. Twelve hours later, two 5 mL solutions were removed from each flask and placed into wells in Cellstar^®^ flat-bottom 24-well plates. The first 5 mL solution, containing approximately 250 J2s, was placed into wells (1 mL per well) just after removal from the flask, observed using an inverted microscope, and categorized as motile or paralyzed (T0). After counting, all J2s were discarded. The second 5 mL solution was rinsed in tap water using a 20 μm sieve, collected in a beaker, and placed in wells (1 mL per well containing approximately 50 J2s). After 12 h (T12), motile and paralyzed J2s were observed using an inverted microscope (100×). J2s were remained in wells, and another two counts were performed after 24 (T24) and 48 (T48) h. No motility regained was evidence of nematicidal activity (permanent death) of the EO. The experiment was carried out twice and every treatment was replicated five times per trial.

### 4.5. Effect of EO and Hydrosol on Egg Differentiation

Eggs of the two species of nematodes were extracted from tomato (*Solanum lycopersicum* cv. Belladona) roots, using the hypochlorite method [32]. The suspension of eggs was placed on a sieve (pore size 38μm), thoroughly rinsed with tap water, and collected into a 100 mL beaker. The suspension of eggs was quantified with the aid of an inverted microscope (100×), and the number of eggs per mL was adjusted and used directly in the bioassays. Essential oil solutions, at 0, 7.8, 15.6, 31.2, 62.5, 125, 250, and 500 μL/L concentrations (in ethanol and Tween-20), and hydrosol dilutions (in distilled water) at 0.01, 0.02, 0.05, 0.1, 0.2, and 0.5 *v/v* concentrations were tested on the development of eggs. The solutions were prepared as previously described. Distilled water was used as a control. Approximately 50 eggs per well, of which 90% were undifferentiated (eggs containing only cells), were exposed to either EO or HL solutions and incubated at 26 ± 1 °C. All plates were covered with aluminum foil to avoid evaporation. For monitoring egg differentiation, eggs were observed on day 0 and day 28, with the aid of an inverted microscope (Zeiss, Germany) at 100× magnification. The experiment was carried out twice, and each treatment was replicated five times.

### 4.6. Hatching Inhibition as Affected by the Presence of EO and Hydrosol

Mature egg masses (dark yellow color) of *Meloidogyne incognita* and *M. javanica* were handpicked using sterilized forceps from roots free of soil. Egg masses were placed in small plastic extraction trays made using 6 cm diameter Petri dishes (one egg mass per extracting tray). Solutions of *C. cyminum* essential oil (7.8, 15.6, 31.2, 62.5, 125, 250, and 500 μL/L) or hydrosol (0.01, 0.02, 0.05, 0.1, 0.2, and 0.5 *v*/*v*) were added to each extracting tray to cover egg masses. Egg masses were maintained for 7 days, and all J2s hatched in test solutions were collected and counted. Then, test solutions were removed by washing them with tap water and placed in new extracting trays filled with clean water. Extracting trays were covered with aluminum foil to avoid evaporation and placed in an incubator at 26 ± 1 °C. After 7 days, all J2s in Petri dishes were counted; then, they were discarded, and the water was substituted with fresh water. The same procedure was repeated every 7 days. The experiment was terminated when J2s no longer emerged, 35 days later. Then, every egg mass was handpicked from Petri dishes, placed in a drop of water on a glass slide, and squashed using a coverslip; all unhatched eggs were counted using an inverted microscope to obtain the total number of eggs at the beginning of the hatching test (hatched + unhatched eggs). The percentage hatch was calculated as the ratio between hatched J2s and the total number of eggs. The experiment was carried out twice, and each treatment was replicated five times per trial.

### 4.7. Effect of EO and Hydrosol on Juveniles in Soil

A soil collected at Gargalianoi village (Peloponnese, Greece) was autoclaved to kill any plant parasitic nematodes and then used in the experiment to verify the effects of C. *cyminum* EO and HL against *M.* javanica J2s. EO solutions at 7.8, 15.6, 31.2, 62.5, 125, 250, and 500 μL/L concentrations, and HL dilutions (in distilled water) at 0.01, 0.02, 0.05, 0.1, 0.2 and 0.5 *v/v* concentrations were tested on J2s in soil. Five plastic pots (9 cm deep and 5 cm diameter) per treatment were filled with 350 g of the treated soil, and 60 mL of the test solutions were applied in each pot. After 2 h, 1 mL of a suspension containing 500 J2s was used for inoculation by making one hole in the center of each pot. Pots were covered with aluminum foil to avoid water evaporation and maintained at 25 ± 1 °C for 24 h to be sure that juveniles were in contact with the chemicals. Then, a seedling of tomato (cv Belladonna) at the four-leaf stage was transplanted in the center of each pot. All plants were placed in a growth room at 25 ± 1 °C with a 16 h photoperiod; 28 days later, they were uprooted, stems were removed, and roots were gently washed free of soil. Roots were stained using acid fuchsin as described in Byrd et al. [33]. Roots were then washed in water and placed in vials containing equal volumes of glycerol and distilled water. Female nematodes were counted in the whole root system of each plant using a stereoscopic microscope at 12.5× magnification. All treatments were replicated five times per trial, while the experiment was performed twice.

### 4.8. Statistical Analysis

One-way analysis of variance (ANOVA) was performed using the general linear model (GLM) in SAS (SAS University Edition). Treatment means were compared using Tukey’s honestly significant difference (HSD) test at *p* < 0.05. In cases where no variation was revealed after ANOVA between the two experiments, data were combined and analyzed together.

## 5. Conclusions

In conclusion, our work showed that EO and HL extracted from *C. cyminum* seeds can control *M. incognita* and *M. javanica*. Our results underlined the permanent paralysis activity against J2s along with the inhibition of egg differentiation and hatching. On the basis of these results, EO and HL extracted from *C. cyminum* seeds could be favorably considered in the use of root-knot nematode control strategies, although further studies are necessary to determine the most efficient application rate in field conditions with different soils, crops, and application times.

## Figures and Tables

**Table 1:** Chemical composition of the essential oil (EO) and hydrosol (HL) of *Cuminum cyminum*.

No	RRI *	Compound	*C. cyminum* EO	*C. cyminum* HL
1	974	*β*-Pinene	5.2	- ^#^
2	997	*α*-Phellandrene	0.7	-
3	1020	*p*-Cymene	6.6	-
4	1049	*γ*-Terpinene	11.1	-
5	1136	Sabinol ^&^	-	0.3
6	1172	4-Terpineol	0.4	1.8
7	1183	*p*-Cymen-8-ol	-	0.2
8	1187	*α*-Terpineol	1	1.3
10	1238	Cumin aldehyde	26.5	31.5
11	1282	*α*-Terpinen-7-al	12.8	20.9
12	1286	*γ*-Terpinen-7-al	34.9	42
13	1321	*p*-Mentha-1,4-dien-7-ol	-	1.3
14	1462	*α*-Acoradiene	0.1	-
15	1592	Carotol	0.1	-
		Total (%)	99.4	99.3
		Grouped compounds		
		Hydrocarbon monoterpenes (HMs)	23.6	-
		Oxygenated monoterpenes (OMs)	75.6	99.3
		Hydrocarbon sesquiterpenes (HSs)	0.1	-
		Oxygenated sesquiterpenes (OSs)	0.1	-

* RRI: relative retention index obtained on TR-5MS column using a series of *n*-alkanes (C8–C24); ^#^ not detected; ^&^ correct isomer not identified.

**Table 2:** Motility percentages of *Meloidogyne incgonita* second-stage juveniles (J2s) after immersion in *Cuminum cyminum* seed essential oil at 0, 7.8, 15.6, 31.2, 62.5, 125, 250, and 500 μL/L concentrations for 24, 48, and 96 h exposure times.

Concentration (μL/L)	Incubation Period (h)					
	24		48		96	
	Experiment 1	Experiment 2	Experiment 1	Experiment 2	Experiment 1	Experiment 2
	Paralyzed J2s (%)		Paralyzed J2s (%)		Paralyzed J2s (%)	
0	1.0 e *	0.7 e	4.6 e	0.7 e	9.7 c	11.6 d
7.8	2.9 de	6.5 de	12.7 d	9.8 d	94.4 b	53.5 c
15.6	6.3 d	7.1 d	21.1 c	12.7 d	93.3 b	61.0 b
31.2	12.0 c	13.8 c	29.9 b	19.0 c	95.4 b	60.0 bc
62.5	88.4 b	55.0 b	99.0 a	71.5 b	100.0 a	100.0 a
125	99.6 a	99.3 a	100.0 a	100.0 a	100.0 a	100.0 a
250	100.0 a	100.0 a	100.0 a	100.0 a	100.0 a	100.0 a
500	100.0 a	100.0 a	100.0 a	100.0 a	100.0 a	100.0 a

* Data flanked in each column by the same letter are not statistically different according to Tukey’s honestly significant difference (HSD) test at *p* < 0.05.

**Table 3:** Motility percentages of *M. javanica* J2s after immersion in *Cuminum cyminum* seed essential oil at 0, 7.8, 15.6, 31.2, 62.5, 125, 250, and 500 μL/L concentrations for 24, 48, and 96 h exposure times.

Concentration (μL/L)	Incubation Period (h)					
	24		48		96	
	Experiment 1	Experiment 2	Experiment 1	Experiment 2	Experiment 1	Experiment 2
	Paralyzed J2s (%)		Paralyzed J2s (%)		Paralyzed J2s (%)	
0	0.0 d *	0.0 c	0.0 d	0.0 c	0.8 d	0.0 c
7.8	0.0 d	0.0 c	0.8 d	0.5 c	27.0 b	28.7 b
15.6	0.5 d	0.0 c	1.8 d	0.0 c	22.5 b	24.5 b
31.2	4.8 c	0.0 c	7.0 c	0.7 c	12.7 c	23.5 b
62.5	97.7 b	91.0 b	97.7 b	94.1 b	100.0 a	100.0 a
125	100.0 a	100.0 a	100.0 a	100.0 a	100.0 a	100.0 a
250	100.0 a	100.0 a	100.0 a	100.0 a	100.0 a	100.0 a
500	100.0 a	100.0 a	100.0 a	100.0 a	100.0 a	100.0 a

* Data flanked in each column by the same letter are not statistically different according to Tukey’s HSD test at *p* < 0.05.

**Table 4:** Motility percentages of *Meloidogyne incgonita* J2s after immersion in *Cuminum cyminum* seed hydrosol at 0, 0.01, 0.02, 0.05, 0.1, 0.2, and 0.5 *v/v* concentrations for 24, 48, and 96 h exposure times.

Dilution (*v/v*)	Incubation Period (h)					
	24		48		96	
	Experiment 1	Experiment 2	Experiment 1	Experiment 2	Experiment 1	Experiment 2
	Paralyzed J2s (%)		Paralyzed J2s (%)		Paralyzed J2s (%)	
0	0.0 f *	0.0 c	9.5 e	0.0 d	22.5 c	0.0 d
0.01	15.1 e	0.0 c	46.2 d	9.5 c	92.9 b	38.1 c
0.02	18.8 e	2.2 c	52.3 d	7.2 c	93.5 b	45.5 c
0.05	35.9 d	30.1 b	65.1 c	36.4 b	92.5 b	71.6 b
0.1	52.6 c	98.8 a	66.1 c	99.9 a	91.6 b	100.0 a
0.2	73.4 b	100.0 a	88.1 b	100.0 a	95.3 b	100.0 a
0.5	100.0 a	100.0 a	100.0 a	100.0 a	100.0 a	100.0 a

* Data flanked in each column by the same letter are not statistically different according to Tukey’s HSD test at *p* < 0.05.

**Table 5:** Motility percentages of *Meloidogyne javanica* J2s after immersion in *Cuminum cyminum* seed hydrosol at 0, 0.01, 0.02, 0.05, 0.1, 0.2, and 0.5 *v/v* concentrations for 24, 48, and 96 h exposure times.

Dilution (*v/v*)	Incubation Period (h)					
	24		48		96	
	Experiment 1	Experiment 2	Experiment 1	Experiment 2	Experiment 1	Experiment 2
	Paralyzed J2s (%)		Paralyzed J2s (%)		Paralyzed J2s (%)	
0	0.0 d *	0.6 d	1.3 e	0.6 c	8.8 f	1.7 d
0.01	1.4 d	0.6 d	18.2 d	10.8 b	67.3 d	46.5 b
0.02	1.6 d	5.1 c	15.7 d	14.0 b	46.3 e	39.0 c
0.05	20.0 c	93.9 b	28.5 c	97.0 a	75.9 c	98.8 a
0.1	74.4 b	99.3 a	81.0 b	99.3 a	91.2 b	100.0 a
0.2	96.7 a	100.0 a	97.4 a	100.0 a	98.6 a	100.0 a
0.5	100.0 a	100.0 a	100.0 a	100.0 a	100.0 a	100.0 a

* Data flanked in each column by the same letter are not statistically different according to Tukey’s HSD test at *p* < 0.05.
